# Breeding, Genetics, and Genomics Approaches for Improving Fusarium Wilt Resistance in Major Grain Legumes

**DOI:** 10.3389/fgene.2020.01001

**Published:** 2020-10-23

**Authors:** Uday Chand Jha, Abhishek Bohra, Shailesh Pandey, Swarup Kumar Parida

**Affiliations:** ^1^ICAR-Indian Institute of Pulses Research, Uttar Pradesh, India; ^2^Forest Protection Division, Forest Research Institute, Dehradun, India; ^3^National Institute of Plant Genome Research, New Delhi, India

**Keywords:** Fusarium wilt, genomics, molecular markers, genetic variability, grain legume

## Abstract

Fusarium wilt (FW) disease is the key constraint to grain legume production worldwide. The projected climate change is likely to exacerbate the current scenario. Of the various plant protection measures, genetic improvement of the disease resistance of crop cultivars remains the most economic, straightforward and environmental-friendly option to mitigate the risk. We begin with a brief recap of the classical genetic efforts that provided first insights into the genetic determinants controlling plant response to different races of FW pathogen in grain legumes. Subsequent technological breakthroughs like sequencing technologies have enhanced our understanding of the genetic basis of both plant resistance and pathogenicity. We present noteworthy examples of targeted improvement of plant resistance using genomics-assisted approaches. In parallel, modern functional genomic tools like RNA-seq are playing a greater role in illuminating the various aspects of plant-pathogen interaction. Further, proteomics and metabolomics have also been leveraged in recent years to reveal molecular players and various signaling pathways and complex networks participating in host-pathogen interaction. Finally, we present a perspective on the challenges and limitations of high-throughput phenotyping and emerging breeding approaches to expeditiously develop FW-resistant cultivars under the changing climate.

## Introduction

Grain legumes being a rich source of dietary proteins and essential minerals serve as one of the vital components of human food (Graham and Vance, 2003; Bohra et al., 2015). Besides, grain legumes also supply essential micronutrients to the human population for combating increasing malnutrition related problems worldwide (Mudryj et al., 2014). Global legume production is severely challenged by a variety of fungal diseases (Kaiser et al., 2000), of which wilt caused by *Fusarium oxysporum* is one of the most destructive (Wade, 1929; Haware et al., 1978; Armstrong and Armstrong, 1981; Reddy et al., 1990; Kraft et al., 1998; Smith et al., 1999; Fall et al., 2001). It ranks fifth among top 10 fungal pathogens of research and economic importance (Dean et al., 2012). The fungus is soil-borne, and occurs as pathogenic (plant, animal, and human) as well as non-pathogenic strains (Leslie and Summerell, 2006). The pathogenic strains have been assigned to *forma specialis* (plural: *formae speciales*), abbreviated f. sp. (plural ff. spp.) based on host specificity (Baayen et al., 2000; Lievens et al., 2008). For instance, chickpea (*Cicer arietinum* L.) is affected by *F. oxysporum* f. sp. *ciceris* (*Foc*). Interestingly, *Foc* can also invade root tissues of other grain legumes, such as *Vicia faba*, *Lens culinaris*, *Pisum sativum*, and *Cajanus cajan* without causing external symptoms (Jiménez-Díaz et al., 2015). Presently Index Fungorum^1^ lists 124 special forms, whereas MycoBank^2^ lists 127 special forms of *F. oxysporum*. Following entry of Fusarium wilt (FW) pathogen through plant root, its colonization in the vascular system disrupts plant root-water continuum, leading to wilting symptoms and death of plant (Schäfer, 1994). Upon exposure to the wilt pathogen, plants recognize pathogen-associated molecular patterns (PAMPs) through their receptor protein pattern recognition receptors (PRRs) known as pattern triggered immunity (PTI) and pathogen effector triggered immunity (ETI), two important mechanisms for averting FW attacks (Zipfel and Robatzek, 2010; Langner et al., 2018). Among the various strategies devised to control FW disease, developing host plant resistance through breeding remains the most straightforward, economic, and sustainable approach. Therefore, physiological pathotypes or races, the subspecific ranks applied to *formae speciales* based on cultivar specificity, are extremely important to breeders for resistance breeding. However, race labels have been incoherent (Gerlagh and Blok, 1988; Correll, 1991; Kistler, 1997; Fourie et al., 2011) as numerous different race designation systems have been applied (Gabe, 1975; Risser et al., 1976; Armstrong and Armstrong, 1981) that created confusion (Kistler, 1997). Nevertheless, recent technological advances in molecular biology helped to overcome many of these hurdles. For instance, sequence-characterized amplified region (SCAR) markers facilitated race identification (Lievens et al., 2008; Epstein et al., 2017; Gilardi et al., 2017). However, laborious and prolonged pathogenicity tests are still required for the identification of new emerging races and resistance testing of the newly developed cultivars against the known races (Epstein et al., 2017; Gilardi et al., 2017). Understanding the genetic makeup of host plant resistance is crucial in this regard. In grain legumes, initial studies based on Mendelian genetics have elucidated a number of gene(s)/genetic determinants underlying resistance against FW (Wade, 1929; Hare et al., 1949; Upadhyaya et al., 1983a, b; Pandey et al., 1996; Coyne et al., 2000; Cross et al., 2000; Fall et al., 2001; Kotresh et al., 2006). Subsequent advances in genomics accelerated molecular mapping of FW resistance gene(s)/QTLs. To this end, availability of whole genome sequences of both plant and pathogen has shed deep insights into the host-pathogen relationship through leveraging comparative genomics approach (Varshney et al., 2012, 2013; Schmutz et al., 2014; Williams et al., 2016; Srivastava et al., 2018; Kreplak et al., 2019; Lonardi et al., 2019). In this review, we discuss the targeted improvement of plant resistance against FW using genomics-assisted approaches. The contributions of functional genomics toward delineating genomic regions/candidate gene(s) responsible for FW resistance in major grain legumes are highlighted. The potential of new omics approaches is discussed with respect to molecular players, signaling pathways, and complex networks underlying host-pathogen interactions. Finally, we present a perspective on high-throughput disease screening and emerging novel breeding techniques for developing FW-resistant cultivars under the changing climate.

## Mode of FW Infection and Possible Mechanism of Host Plant Resistance Against FW

*Fusarium oxysporum* is considered saprophytic because of its ability to survive on soil organic matter for several years (Alabouvette et al., 1993). The fungus survives in soil through producing chlamydospores that may serve as a reservoir of inoculums (Schippers and Van Eck, 1981). Subsequent to penetrating plant root epidermis, the pathogen invades the xylem vessels to cause wilting symptoms (Srinivas et al., 2019). The complete molecular mechanism of FW pathogenesis remains to be elucidated (Rep and Kistler, 2010). Genome-wide transcriptome profiling of conidial germination of one of the most virulent Indian races (race 1) of *Foc* has revealed germination-related genes and families of genes encoding secreted effectors, cell wall/pectin-degrading enzymes, metabolism related enzymes, transporters, and peptidases. Importantly, qRT-PCR confirmed the up-regulation of metabolism related enzymes during early infection, whereas up-regulation of most transporters and secondary metabolites important for tissue colonization and pathogenicity was confirmed during later stages (Sharma et al., 2016b).

Following establishment of the pathogen on plant roots, root penetration, and hyphal propagation of the FW pathogen causes a compromise in the host defense system (for detail see Rep and Kistler, 2010; Srinivas et al., 2019). At molecular level, the fungal pathogen recognizes a particular host and produces a range of cell wall-degrading enzymes including cellulases, pectinases, polygalacturonases, etc., in response to host plant derived hydrolases (viz., chitinase, glucanases) (Michielse et al., 2009; Swarupa et al., 2014; Husaini et al., 2018). Besides, the FW pathogen is also known to produce various mycotoxins/phytotoxins viz., fusaric acid (FSA), beauvericin, and enniatins in banana (López-Díaz et al., 2017; Liu et al., 2020; Shao et al., 2020). In parallel, the attacking pathogen integrates various signal transduction pathways mediated by mitogen activated protein (MAP) kinase cascades that transduce the signal downstream to the intracellular targets in response to the signal perceived by various receptors at cell surface during host infection (Widmann et al., 1999; Husaini et al., 2018). Thus, MAP plays a key role in regulating FW pathogenicity. Several genes are known to regulate host colonization and pathogenicity, which include *FWO1* (Inoue et al., 2002), *ClcI* (Cañero and Roncero, 2008), *chitin synthase V*, *DCW1*, *mannose- 6- phosphate isomerase* gene, *FOXG_11097* (Michielse et al., 2009), *XlnR* (Calero-Nieto et al., 2007), secreted in xylem (SIX) protein encoding genes (Thatcher et al., 2016), *SIX1, SIX6*, and *FTF1* genes (NinÞo-Saìnchez et al., 2015) (for details see Husaini et al., 2018). Thus, successful disease occurrence by the FW pathogen demands a compromise in the plant defense system.

**FIGURE 1:**
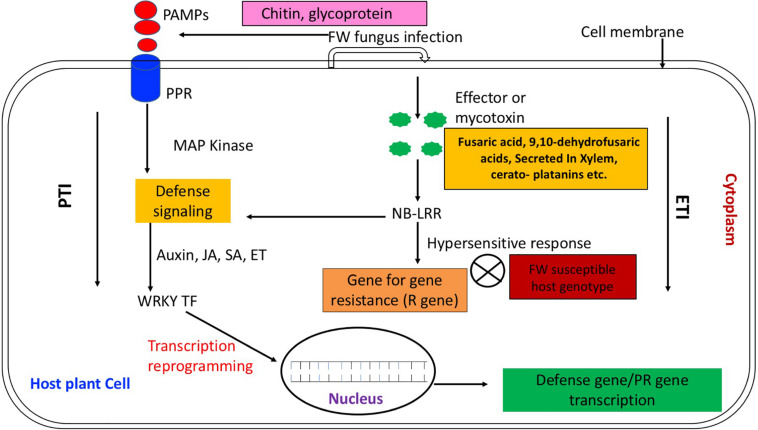
In PTI following the perception of PAMPs by PRRs, plant evokes signaling networks mediated by protein kinases viz., mitogen-activated protein kinases (MAPK) and protein phosphorylation (Nakagami et al., 2005; Boudsocq et al., 2010; Rodriguez et al., 2010; Lanubile et al., 2015). In turn, this is accompanied by activation of multiple TFs genes (via auxin, JA, ET) that ultimately switch on transcription of various defense responsive genes including PR genes (Castillejo et al., 2015). Once the PTI fails, the ETI forms the second line of immune response in which plant defends itself against pathogen attack through encoding immune receptors of the nucleotide binding leucine rich repeat (NB-LRR) class by R genes that enable recognizing the effector molecules (viz., fusaric acid) (Bani et al., 2014) produced by pathogen and initiate hypersensitive response (Zipfel and Robatzek, 2010; Ma and Ma, 2016). In case of susceptible host, the effector molecules remain unrecognized and there is no host pathogen hypersensitive response (Li et al., 2020).

The PTI and the ETI activated by the PAMPs and pathogen effectors, respectively constitute the two tiers of the plant defense system. The PTI, presenting the first line of plant defense, ensues by the recognition of PAMPs *via* the host receptor protein PRRs (Zipfel and Robatzek, 2010; Beck et al., 2012; Lanubile et al., 2015). Following this, the plant evokes oxidative burst and ion influx that transduce the signals to different pathways by triggering down-stream signaling networks mediated by the protein kinases viz., mitogen-activated protein kinases (MAPK) and protein phosphorylation (Nakagami et al., 2005; Pedley and Martin, 2005; Boudsocq et al., 2010; Rodriguez et al., 2010; Bigeard et al., 2015; Lanubile et al., 2015). This is accompanied by the activation of multiple TFs that switch on various defense responsive genes including PR genes and the genes involved in hormone biosynthesis and signaling, protein and sugar metabolism (Castillejo et al., 2015; Lanubile et al., 2015; Kumar et al., 2016). As shown in Figure 1, once the PTI fails, the ETI forms the second line of immune response in which the plant defends itself against pathogen attacks through immune receptors encoded by the nucleotide binding leucine rich repeat (NB-LRR) class of R genes, thus enabling recognition of the effector molecules (Zipfel and Robatzek, 2010; Bigeard et al., 2015; Ma and Ma, 2016). This in turn triggers the plant innate immunity that inhibits the pathogen attack (Dodds and Rathjen, 2010). These resistance genes are overcome by the more virulent races of pathogen; hence, a broad-spectrum and durable host resistance is highly needed (Yin and Qiu, 2019; Li et al., 2020).

## FW Races and Their Effects on Major Grain Legumes

### Chickpea

*Fusarium oxysporum* f. sp. *ciceris* is prevalent in all major chickpea growing countries including India, Iran, Peru, Syria, Ethiopia, Mexico, and the United States (Nene et al., 1989; Halila and Strange, 1996). Up to 40% yield losses have been reported in chickpea due to FW, and the disease may lead to complete crop failure under congenial environment (Haware et al., 1978, 1986; Sharma et al., 2014). In recent times, FW has emerged as a severe threat to chickpea production because of a shift in chickpea area from long prevailing cool season of northern region to warm regions of southern and central India (Sharma and Pande, 2013).

Early wilting is manifested in the form of dull green discoloration within 25 days after sowing accounts for 77–94% yield loss (Jiménez-Díaz et al., 2015). In the case of “late wilt” dropping petioles and yellowing of leaf symptoms appear at podding stage, causing 24–65% yield loss (Jiménez-Díaz et al., 2015). Based on the symptoms produced on host plant, *Foc* races are grouped into two categories. Six races (1A, 2, 3, 4, 5, and 6) cause wilting symptoms (see Table 1), while two races (0 and 1B/C) cause yellowing symptoms in the plant during infection (Haware and Nene, 1982; Jimenez-Diaz et al., 1993; Kelly et al., 1994; Sharma et al., 2004). Among these *Foc* races, races 2, 3, and 4 are prevalent in India. Races 0, 1B/C, 5, and 6 are reported in Mediterranean and the United States, whereas the race 1A occurs in India, the United States, and the Mediterranean region (Jimenez-Diaz et al., 1993; Jiménez-Gasco et al., 2001; Landa et al., 2006). Gurjar et al. (2009) classified race 3 as *Fusarium proliferatum* based on phylogenetic analysis of translation elongation factor 1 alpha (TEF1-alpha) sequence data. The study demonstrated the reliable identification of Indian *Foc* races using a diverse DNA marker system. Earlier, Jiménez-Gasco and Jiménez-Díaz (2003) developed SCAR markers from race-specific random amplified polymorphic DNA (RAPD) markers for the identification of *Foc* and its pathogenic races. Change in the race scenario of *Foc* has been reported in India. Seventy *Foc* isolates, collected from 13 states and four crop cultivation zones of India, grouped into eight races based on their response to differential cultivars of chickpea. Further, characterization with four different molecular markers (RAPD, universal rice primers, SSR, and ISSR) grouped the isolates into eight clusters, which partially corresponded to the chickpea-growing zones and the racial distribution of the pathogen (Dubey et al., 2012). The presence of 47 isolates of different *Foc* races into one vegetative compatibility group (VCG) suggested its monophyletic origin (Nogales Moncada et al., 2009).

**TABLE 1:** List of FW races and their effects on various grain legumes.

Crop	Causal organism	Races	Symptoms	% Yield loss	References
Chickpea	*Fusarium oxysporum* f.sp. *ciceri* (*Foc*)	Eight races (0, 1B/C, 1A, 2, 3, 4, 5, and 6)	Destruction of vascular bundle that leads to inflicting plant water relation, drooping of petioles, rachis and leaflets; Races 0 and 1B/C cause yellowing syndrome	10–40% and even 100% under favorable condition	Haware and Nene, 1982; Jimenez-Diaz et al., 1993; Kelly et al., 1994; Sharma and Muehlbauer, 2007; Castro et al., 2010; Jiménez-Díaz et al., 2015
Pea	*Fusarium oxysporum* Schl. f. sp. *pisi* Snyd. and Hans. (*Fop*)	Four races (1, 2, 5, and 6)	Gray-green discolored foliage, thickening of basal internodes, downward curling of the leaves from the base to the apex, chlorosis with unilateral wilting, yellow to orange discoloration in the vascular tissue wilting and death of the entire aerial part race 1 and race 5 cause complete death of plant	up to 100%	Wade, 1929; Hare et al., 1949; Coyne et al., 2000; Haglund and Kraft, 2001; Jain et al., 2015
Cowpea	*Fusarium oxysporum* f.sp. *tracheiphilum* (*Fot*)	Four races (1, 2, 3, and 4)	Wilting and leaf chlorosis and stunting the entire plant typical symptoms evident during the flowering and early pod development stages causing high mortality in the affected areas with severe overall yield loss	up to 100%	Hare, 1953; Patel, 1985; Smith et al., 1999; Davis and Frate, 2007; Pottorff et al., 2014
Pigeon pea	*Fusarium udum* Butler	–	–	30–100%	Reddy et al., 1990; Okiror, 2002
Common bean	*Fusarium oxysporum* (Schlecht.) f. sp. *phaseoli* Kendrick & Snyder (*FOP*)	Two race (2, 4)	Phloem blockage; chlorosis and wilt in older leaves wilting of younger leaves, and necrosis of the apex and vascular tissue Vascular tissues turn a red-brown color, shortening of plant lifecycle and plant death	up to 100%	Abawi and Pastor-Corrales, 1990; Buruchara and Camacho, 2000; Fall et al., 2001; Xue et al., 2015
Lentil	*Fusarium oxysporum* f. sp. *lentis* (*Fol*)	Eight races/pathotypes	Stunting, wilting, internal vascular discoloration in lower stem, wilting symptoms appear in seedling, flowering and pod stages	up to 100%	Kannaiyan and Nene, 1976; Agrawal et al., 1991; Erskine and Bayaa, 1996; Tosi and Cappelli, 2001; Hiremani and Dubey, 2018
Faba bean	*Fusarium oxysporum* f. *fabae* (*FOF*)	–	–	–	Abdel Rehim, 1962

### Cowpea

*Fusarium oxysporum* f. sp. *tracheiphilum* (*Fot*), a soil-borne fungus, severely challenges cowpea production (Armstrong and Armstrong, 1981). The disease is prevalent in all cowpea growing countries across the world including the United States, central valley of California (Hare, 1953; Smith et al., 1999), Australia (Summerell et al., 2011), Brazil and Nigeria (Armstrong and Armstrong, 1980; Assunção et al., 2003). Four *Fot* races (1, 2, 3, and 4) have been identified based on their pathogenic reactions to differential cowpea genotypes. Worldwide occurrence of race 3 has been reported among various *Fot* races (Hare, 1953; Smith et al., 1999). Broad patches of diseased cowpea plants with visible symptoms of chlorosis, wilting and stunting at the seedling stage or during flowering and early pod development result in high mortality and significant yield loss (Smith et al., 1999; Pottorff et al., 2012; see Table 1).

### Common Bean

Wilt of common bean caused by *F. oxysporum* f. sp. *phaseoli* (*FOP*) results in substantial yield loss. *FOP* is commonly found in various common bean growing countries across the United States, China, Africa, and Latin America (Harter, 1929; Buruchara and Camacho, 2000). Elena and Papas (2002) reported that most of the isolates among 27 *FOP* isolates from Greece belonged to the same VCG. Earlier, VGC and molecular analysis of 128 *FOP* isolates collected from the field of El Barco de Avila in Spain suggested a difference between *FOP* isolates of Spain and America thus, supporting its pathogenic evolution (Alves-Santos et al., 1999).

The *FOP* invades plants through penetrating roots and colonizes the cortical cell. The hyphae move toward vascular parenchyma cells accompanied by a collapse of the xylem vessel and subsequent disruption of the water uptake from the roots (NinÞo-Saìnchez et al., 2015; Garces-Fiallos et al., 2017). Recent histological evidences support faster colonization of *FOP* in the susceptible genotypes in comparison to the resistant genotypes (Garces-Fiallos et al., 2017). A previous study by NinÞo-Saìnchez et al. (2015) explained the differential pattern of host plant colonization of highly virulent and weakly virulent strains of *FOP* based on expression analysis of different virulence genes viz., *FTF1, SIX1*, and *SIX6*. The infected plant displays wilting of older leaves followed by younger leaves and necrosis of the apex part (Fall et al., 2001; NinÞo-Saìnchez et al., 2015; Batista et al., 2017; Garces-Fiallos et al., 2017), internal stem discoloration (Batista et al., 2016), leading to death of the plant (Fall et al., 2001; Batista et al., 2017).

### Lentil

*Fusarium oxysporum* f. sp. *lentis* (*Fol*) is the causal agent of wilt disease in lentil (Erskine and Bayaa, 1996; Bayaa et al., 1998; Tosi and Cappelli, 2001). In India, 0.7–9.3% mortality has been reported at reproductive stage based on a survey of 116 districts of nine lentil growing states (Chaudhary et al., 2010). Similarly, severe disease incidence was recorded in 21 locations of Pakistan (Rubab et al., 2014). The pathogen survives in plant debris/soil and infects the host through the roots. The ability of the pathogen to survive in soil for long periods through chlamydospores further aggravates the situation. Evidence also suggests transmission of the pathogen through seeds (Erskine et al., 1990). In Algeria, based on the pathogenic variation of 32 isolates of *Fol*, Belabid et al. (2004) reported homogeneous behavior of Algerian *Fol* isolates with no variations in virulence and the existence of one *Fol* race. Although resistant or moderately resistant wilt varieties have been developed, variable responses of these varieties across agro-ecological niches imply the presence of high pathogen variability (Naimuddin and Chaudhary, 2009). No pathotypes within this *formae specialis* were identified until the study of Pouralibaba et al. (2016). Upon inoculation of 28 lentil resistant accessions with six *Fol* isolates with different geographical origins, a highly significant isolate × accession interaction resulted in the identification of four accessions as a putative differential set. The virulence pattern of 52 *Fol* isolates from Iran, Syria, and Algeria allowed the identification of seven pathotypes (1–7). Similarly, in India, Hiremani and Dubey (2018) identified eight races/pathotypes of the pathogen based on resistant and susceptible reactions on a set of differential cultivars. These studies pave the way for developing race specific wilt resistant lentil cultivars and help in the identification of new races from different lentil growing areas worldwide.

### Pea

Wilt caused by *F. oxysporum* f. sp. *pisi* (*Fop*), is among the most significant yield reducers in pea growing areas (Kraft et al., 1981; Infantino et al., 2006; Rubiales et al., 2015). Among the four races (1, 2, 5, and 6) of *Fop*, races 1 and 5 are the most destructive, causing death of the plant (Jain et al., 2015). However, race 2 remains less pathogenic and wilting symptom appears in post podding stage. Based on VCG, isolates of *Fop 1* and *Fop 6* remained in a single VCG, whereas *Fop5* belonged to a second VCG and *Fop2* were present in another two VCGs (Kraft, 1994). *Fop* infects the root and interferes with the plant water movement, resulting in wilting symptoms (see Table 1). The other notable symptoms of wilt in pea include gray-green discoloration of foliage, chlorosis with unilateral wilting, orange discoloration in the vascular tissue (Jain et al., 2015).

### Pigeonpea

Pigeonpea wilt is caused by *Fusarium udum*, a soil and seed borne pathogen, and the disease is reported to cause up to 100% yield loss in the susceptible cultivars (Reddy et al., 1990; Okiror, 2002). Occurrence of FW is reported predominantly in north and central parts of India, Kenya, and Malawi, and also in Ghana, Tanzania and Uganda (Nene, 1980). The annual losses caused by FW in pigeonpea have been estimated to be United States $71 million and 470,000 tons of grain in India and 30,000 tons of grain in Africa (Saxena et al., 2017).

### Faba Bean

Faba bean cultivation is severely affected by wilt [*F. oxysporum* f. *fabae* (*FOF*)] worldwide (Abdul Wahid et al., 1998; Dong et al., 2014). This disease was first reported by Abdel Rehim (1962) in Egypt.

## Plant Genetic Resources for FW Resistance

Efforts to control FW disease with chemical agents have met with limited success. In view of the hazardous nature of fungicides to the environment, developing host plant resistance presents the most durable, economic and ecofriendly means to minimize the FW loss in grain legumes (Saxena, 2008; Jain et al., 2015). Therefore, harnessing the variation in plant traits that impart FW resistance could substantially improve the resistance level of various grain legumes. Considerable genetic variability has been observed in chickpea genotypes for FW response (Haware et al., 1992; Ali et al., 2002). Identification of resistant sources of FW in both kabuli (ICCV 2 and UC 15) and desi types (FLIP 85-20C, FLIP 85-29C, and FLIP 85-30C) by Ali et al. (2002) was consistent with the earlier report of Jimeìnez-Diìaz et al. (1991). More recently, Sharma et al. (2019) identified several chickpea FW resistant genotypes viz., ICCV 98505, ICCV 07105, ICCV 07111, ICCV 07305 based on GGE biplot analysis (see Table 2). Previously, Kumar et al. (1985) developed four kabuli resistant genotypes (ICCV 2, ICCV 3, ICCV 4, and ICCV 5) through a pedigree method. Recent advancements in genomic technologies in grain legumes have provided crop breeders with a set of more efficient tools for resistance breeding. Consequently, successful examples of genomics-assisted trait improvement for abiotic and biotic stresses are now available in legume crops (Bohra et al., 2014a, b; Varshney et al., 2015). In chickpea, a marker-assisted back crossing (MABC) scheme has allowed targeted transfer of genomic regions conferring FW resistance (*foc4*) from WR 315 to Annigeri 1 and JG 74, two elite yet FW-sensitive elite chickpea cultivars (Mannur et al., 2019). Thus, MABC derived products in chickpea such as Super Annigeri 1 and JG 74315-14 showed an 8% increase in yield and disease resistance over Annegiri and 53.5% increase in yield over JG74, respectively (Mannur et al., 2019). Likewise, genomic regions underlying *foc1* and *foc*2 resistance were transferred from JG 315 to C 214 (Varshney et al., 2014) and from Vijay to Pusa 256 (Pratap et al., 2017) using MABC. The MABC-bred lines carrying favorable alleles such as ICCX-100175-349-2-2, ICCX-100175-382-4-6, and ICCX-100175-389-3-2 had high to moderate resistance against FW (*foc1*) under field conditions (Varshney et al., 2014). Marker-aided breeding schemes could enable efficient pyramiding of QTL into new cultivars, thus imparting on them durable resistance against multiple FW races. Apart from the cultivated pool, crop wild relatives (CWRs) of chickpea viz., *Cicer reticulatum*, *Cicer echinospermum, Cicer bijugum*, *Cicer judaicum*, *Cicer pinnatifidum*, and *Cicer cuneatum* have also been identified having traits that confer FW resistance (Nene and Haware, 1980; Kaiser et al., 1994; Singh et al., 1998).

**TABLE 2:** List of grain legume genetic resource contributing to FW resistance.

Crop	Resistance source	Name of the institute	References
Chickpea	ICC 11322 (WR 315)	ICRISAT, Patancheru	Singh et al., 1974
Chickpea	*C. bijugum, C. judaicum, C. pinnatifidum, C. reticulatum, C. echinospermum, and C. cuneatum*	ICRISAT, Patancheru	Nene and Haware, 1980; Singh et al., 1998
Chickpea	ICCV2, ICCV3, ICCV4, and ICCV5 (against race l)	ICRISAT, Patancheru	Kumar et al., 1985
Chickpea	ICC 11322, 14424, and 14433 (against to race l)	ICRISAT, Patancheru	Nene et al., 1989
Chickpea	ICC-2862, -9023, -9032, -10803, -11550, and -11551	–	Haware et al., 1990
Chickpea	FLIP 84-43C (against race 0), ILC-5411, FLIP 85-20C (against race 5), FLIP 85-29C, FLIP 85-30C, ILC-127 (against race 0), ILC-219 (against race 0), ILC-237, ILC-267, and ILC-513 (against race 0)	Santaella, Córdoba, Spain	Jimeìnez-Diìaz et al., 1991
Chickpea	*C. cuneatum, C. judaicum* (PI458559 resistant against race 0), *C. bijugum* (PI458550, PI458552 resistant to race 0, 5), *Cicer canariense* (PI553457 resistant against race 0), *Cicer chorassanicum* (PI458553 resistant against race 0), *C. cuneatum, C. judaicum*, and C. *pinnatifidum* (PI 458555, PI458556 resistant to race 0)		Kaiser et al., 1994
Chickpea	ICCV 2 and UC 15 FLIP 85-20C, FLIP 85- 29C, and FLIP 85-30C	Hudeiba Research Station, Ed-Damer, Sudan	Ali et al., 2002
Chickpea	CA-334.20.4, CA-336.14.3.0, and ICC-14216K (race 5)	–	Navas-Cortes et al., 1998; Castillo et al., 2003
Chickpea	Andoum 1 and Ayala (race 0)	–	Halila and Harrabi, 1990; Landa et al., 2006
Chickpea	Surutato-77, Sonora-80, Tubutama, UC-15 and UC-27, Gavilan	Mexico	Morales, 1986; Buddenhagen et al., 1988; Helms et al., 1992; Sharma et al., 2005
Chickpea	BG-212	India	Sharma et al., 2005
Chickpea	ICC-7520	Iran	Sharma et al., 2005
Chickpea	Annigeri	India	Sharma et al., 2005
Chickpea	ICC 7537 resistant to all races (except race 4)	Ethiopia	Sharma et al., 2005
Chickpea	ICC14194, ICC17109, WR315	ICRISAT, Patancheru	Gaur et al., 2006
Chickpea	CM418-1/01, CM446-1/01, CM499/01, CM499-1/01, CM499-2/01		Shah et al., 2009
	CM554-1/01, CM554-2/01, CM557-2/01		
	CM557-5/01, CM557-6/01, CM557-7/01, CM5578/01 and CM499-5/01		
Chickpea	ICCV 09118, ICCV 09113, ICCV 09115, ICCV 09308, ICCV 09314	ICRISAT, Patancheru	Sharma et al., 2010
Chickpea	ICCV 05527, ICCV 05528, ICCV 96818	ICRISAT, Patancheru	Sharma et al., 2012
Chickpea	Three lines derived from MABC based C 214 × WR 315 cross	ICRISAT, Patancheru	Varshney et al., 2014
Chickpea	ICCVs 98505, 07105, 07111, 07305, 08113, and 93706 (highly resistant)	ICRISAT, Patancheru	Sharma et al., 2019
	ICCVs 08123, 08125, 96858, 07118, 08124, 04514, 08323, and 08117(moderately resistant)		
Chickpea	Digvijay	India	Upasani et al., 2016
Chickpea	SCGP-WR 28, H 10-05, GL 10023, IPC 2006-77, and CSJK 72	IARI, New Delhi, India	Dubey et al., 2017
Chickpea	Super annigeri and Improved JG74 (resistant against foc4)	ICRISAT, Patancheru	Mannur et al., 2019
		ARS-Kalaburagi	
		JNKVV, Jabalpur	
Pigeonpea	ICP 9145	ICRISAT, Patancheru	Reddy et al., 1995
Pigeonpea	IC0574574	IIPR, Kanpur	Singh et al., 2011b
Pigeonpea	ICPL 20109, ICPL 20096, ICPL 20115, ICPL 20116, ICPL 20102, ICPL 20106, and ICPL 20094	ICRISAT, Patancheru	Sharma et al., 2016a
Pigeonpea	BDN-2004-1, BDN-2001-9, BWR-133, and IPA-234	–	Singh I. P. et al., 2016
Cowpea	CB46, CB3, 7964, and 8514	University of California, Riverside (UCR)	Roberts et al., 1995
Cowpea	California Blackeye 27, California Blackeye 46, California Blackeye 50 (Fot race 3)	–	Ehlers et al., 2000, 2009
Cowpea	CB27, 524B	University of California, Riverside (UCR)	Muchero et al., 2009
Common bean	HF 465-63-1	–	Pastor-Corrales and Abawi, 1987
Common bean	RWR 950 and G 685	–	Buruchara and Camacho, 2000
Common bean	CAAS260205	Yunnan, China	Xue et al., 2015
Pea	J1412, JI1760, P633 (*P. sativum* ssp. *arvense*), P42(*P. sativum* ssp. *elatius*) against Fop race 2	–	Bani et al., 2012, 2018a
Lentil	ILL 422, ILL 813, ILL 1220, ILL 1462, ILL 2313, and ILL 2684	ICARDA, Syria	Sarker et al., 2001
Lentil	ILWL 79 and ILWL 113 of *L. culinaris* ssp. *orientalis*	Tel Hadya farm, Northern Syria	Bayaa et al., 1995
	ILWL 138 of *L. nigricans* ssp. *ervoides*		
Lentil	81S15, FLIP2007-42 L and FLIP2009-18 L	–	Mohammadi et al., 2012
Lentil	BGE016363, BGE025720, BGE032290, and BGE040548	–	Pouralibaba et al., 2015
Lentil	PL101 and L4076	AICRP, India	Parihar et al., 2017
Faba bean	Assiut-215, Roomy-3, Marut-2, and Giza-2	Assiut University, Egypt	Mahmoud and Abd El-Fatah, 2020

Studies have shown significant genetic variability for *Fol* resistance in lentil (Bayaa et al., 1995; Pouralibaba et al., 2015).

Studies have shown significant genetic variability for *Fol* resistance in lentil (Bayaa et al., 1995; Pouralibaba et al., 2015). Screening in both controlled and field conditions led to the identification of three promising lines viz., 81S15, FLIP 2007-42 L, and FLIP 2009-18 L (Mohammadi et al., 2012) and BGE040548, BGE019708, BGE022526, BGE025720 (Pouralibaba et al., 2015) exhibiting *Fol* resistance. Recently, GGE biplot analysis has revealed two lentil genotypes PL101 and L4076 as promising sources for *Fol* resistance (Parihar et al., 2017). Similarly, resistant genotypes ILWL 79 and ILWL 113 (*L. culinaris* ssp. *orientalis*) and ILWL 138 (*Lens nigricans* ssp. *ervoides*) were screened against *Fol* from 219 wild lentils (Bayaa et al., 1995). Interspecific cross between ILL10829 (*L*. *culinaris* subsp. *culinaris*) and ILWL30 (*Lens ervoides*) has also revealed substantial genetic variability for *Fol* resistance (Singh et al., 2017).

Previously, based on glasshouse screening, Doling (1964) identified Delwiche Commando, New Era and New Season genotypes to be resistant to both *Fop1* and *Fop2* races of FW. Likewise, Kraft (1994) identified 74SN5 pea line to be an important source of resistance against all the four races *Fop1*, *Fop2*, *Fop5*, and *Fop6*. Subsequently, screening of a large set of 452 pea accessions collected from 24 countries resulted in the identification of 62 accessions to be resistant against *Fop2* and 39 accessions out of these 62 also possessed resistant to *Fop1* (McPhee et al., 1999). Additionally, PI 344012 a wild progenitor of pea displayed resistance against both *Fop1* and *Fop2* (McPhee et al., 1999). Given the screening of 117 pea genotypes against *Fop1*, *Fop2*, *Fop5*, and *Fop6* under growth chamber, Neumann and Xue (2003) identified Radley and Princess cultivars exhibiting resistant reaction against *Fop2*, *Fop5*, and *Fop6* races. Likewise, a thorough assessment of eighty accessions of *Pisum* spp. against *Fop2* by recording detailed disease scoring based on the various parameters revealed significant genetic variation for *Fop2* disease reaction (Bani et al., 2012). Further the authors reported eleven accessions namely JI 1412, JI 1559, JI 1760, P 23, P 42, P 614, P 627, P 633, P 639, P 650, and P 656 to be resistant against *Fop2* (Bani et al., 2012). Evaluation of 34 pea genotypes against FW under wilt sick plot and artificially controlled conditions allowed for the identification of GP-6, GP-55, and GP-942 as highly resistant and GP-17, GP-48, GP-473, and GP-941 as resistant donors for *Fop* resistance (Shubha et al., 2016).

Considering the common bean earlier, Ribeiro and Hagedorn (1979) identified the Preto Uberabinha common bean cultivar to be resistant to *FOP* based on disease reaction of plant root inoculated with *FOP* microconidia. Subsequently, rigorous screening of 73 climbing and bush type accessions of common bean against *FOP* led to identification of 19 climbing type and 28 bush type accessions to be resistant against *FOP*. Further, two genotypes RWR 950 and G 685 displayed resistance even at higher inoculum density of *FOP* (Buruchara and Camacho, 2000). Based on the restriction of *FOP* xylem tissue colonization, Pastor-Corrales and Abawi (1987) reported Manteigão Fosco 11 genotype to be resistant against *FOP*. Likewise, UFSC-01 genotype was revealed to be resistant against *FOP* by checking the colonization of *FOP* inside the xylem vessel (Garces-Fiallos et al., 2017). Furthermore, availability of high throughput molecular maker based genome wide association study allowed the identification of 14 highly resistant common bean genotypes against *FOP-SP1* race 6 (Leitão et al., 2020).

In pigeonpea, multi-location and multi-year testing of wider sets of germplasm and advanced breeding lines has revealed several promising lines for regular use in breeding programs. The resistant sources resulting from these evaluations include KPL 43, KPL 44, IPAs 16 F, 8 F, 9 F, and 12 F (Singh et al., 2011a) and ICPLs 20109, 20096, 20115, 20116, 20102, 20106, and 20094 (Sharma et al., 2016a; see Table 2). Notably, resistance to wilt is an essential prerequisite for variety identification and release in pigeonpea. Several pigeonpea varieties such as Asha (ICPL 87119), ICP 8863, BSMR 736, TS3R, IPA 203, BDN 708, BDN 711, etc., show considerable level of resistance to *F. udum* (Singh I. P. et al., 2016; Bohra et al., 2017).

In cowpea, genotypes CB46, CB3, 7964, and 8514 were identified as FW resistant based on three years evaluation at two different locations (Roberts et al., 1995). Similarly, genotypes CB46, CB27, and CB50 could serve as donors for developing FW resistant cowpea (Ehlers et al., 2000, 2009; Muchero et al., 2009). Since screening of large germplasm collections for FW resistance remains time consuming, thus availability of molecular markers linked with FW resistant gene(s) could circumvent the traditional screening methods (Ali et al., 2012; Jain et al., 2015).

In faba bean, variation was reported among 16 lines for FW resistance using inter-simple sequence repeat (ISSR), sequence related amplified polymorphism (SRAP) and SSR markers (Mahmoud and Abd El-Fatah, 2020). Based on the disease severity, Assiut-215, Roomy-3, Marut-2, and Giza-2 were found to be promising for FW resistance in faba bean.

## Genetic Basis of Host Plant Resistance Against FW in Grain Legumes

### Inheritance of FW Resistance in Grain Legumes

Initial studies on genetic inheritance of FW resistance in grain legumes relied on Mendelian genetics. Literature in chickpea on FW inheritance suggests its control by major genes (Upadhyaya et al., 1983a, b; Kumar, 1998; Tullu et al., 1999). For example, genetic resistance against *Foc*1 results from the action of three independent loci *h*_1_, *h*_2_, and *h*_3_ (Upadhyaya et al., 1983a, b; Singh et al., 1987a, b). Kumar (1998) also advocated involvement of three separate loci controlling *Foc*2 resistance. Likewise, other researchers have reported digenic inheritance for other races (0 and 2) of *Foc* (Tullu et al., 1999; Rubio et al., 2003). Previously, the digenic (*a, b*) nature of *Foc*2 resistance was also established based on the disease reaction of F_2_ and F_3_ individuals derived from WR 315 × C 104 (Kumar, 1998). Whereas Tekeoglu et al. (2000) and Sharma et al. (2004) reported monogenic inheritance of *Foc 3* and *Foc* 5 resistance. Concerning resistance against *Foc*4, Tullu et al. (1998, 1999) explained its monogenic and recessive nature in the genotype WR 315, however, its recessive and digenic nature was explained in Surutato 77. Likewise, Sharma et al. (2005) reported that the genetic resistance against each race 1A, 2, 3, 4, and 5 was controlled by a single gene. However, the genetic basis of resistance to races 1B/C and 6 is still to be studied.

Classical genetics studies in pea established that resistance to FW (races 1, 2, 5, and 6) was controlled by different genes of a dominant nature (Wade, 1929; Hare et al., 1949; Coyne et al., 2000; Haglund and Kraft, 2001). Resistance against *Fop* races 1, 5, and 6 was conferred by a single dominant gene, however, resistance to race 2 follows a quantitative pattern (Bani et al., 2012; McPhee et al., 2012). A study by Wade (1929) established that the *Fop*1 resistance was controlled by a single dominant gene (*Fw*), which was *s*ubsequently mapped on to LG III (Grajal-Martin and Muehlbauer, 2002).

Research on *FOP* resistance in common bean has shown presence of a single gene (Ribeiro and Hagedorn, 1979; Salgado et al., 1995; Cross et al., 2000; Fall et al., 2001), oligogene (Ribeiro and Hagedorn, 1979; Salgado et al., 1995; Cross et al., 2000; Fall et al., 2001; Batista et al., 2017) and polygenes (Salgado et al., 1995; Cross et al., 2000; Batista et al., 2016, 2017). Considering resistance against *Fop4*, occurrence of a single dominant gene (Salgado et al., 1995; Cross et al., 2000) as well as QTL was proposed (Fall et al., 2001).

Studies on the inheritance of FW resistance in pigeonpea indicate varying patterns such as two complementary genes (Pathak (1970), dominant monogenic (Pawar and Mayee, 1986; Pandey et al., 1996; Kotresh et al., 2006; Karimi et al., 2010), recessive monogenic (Jain and Reddy, 1995; Odeny et al., 2009), one dominant and one recessive gene with dominant suppressive epistatic (Saxena et al., 2012) as well as polygenic inheritance (Pal, 1934). Recent analysis of populations derived from four resistant and four susceptible parents suggested that the FW resistance was governed by one dominant gene each in BDN 2004-1 and BDN 2001-9 in comparison to two duplicate dominant genes in BWR 133 and two dominant complimentary genes in IPA 234 (Singh D. et al., 2016).

In lentil, limited research has been done on understanding the genetic basis of resistance to *Fol*. Kamboj et al. (1990) proposed the presence of five independently segregating genes for *Fol* resistance based on allelic tests of the crosses involving three *Fol* resistant lines (L 234, JL 446, and LP 286) and two susceptible lines (L 9-12 and JL 641). Subsequently, Eujayl et al. (1998) established monogenic dominant inheritance of *Fol* resistance based on F_2__:__4_ progenies [ILL 5588 × L 692–16−l(s)].

### Identification of Resistance Loci and Molecular Marker for FW Resistance in Grain Legumes

DNA marker technologies have facilitated locating/mapping of gene(s) controlling resistance against various races of *Foc* in chickpea (Mayer et al., 1997; Ratnaparkhe et al., 1998; Tullu et al., 1999; Tekeoglu et al., 2000; Winter et al., 2000; Rubio et al., 2003; Sharma et al., 2004; Cobos et al., 2005). Previously, Cobos et al. (2005) reported the *Foc0*_1_/*foc0*_1_ gene on LG 5 flanked by OPJ20600 and TR59 markers. Later, Halila et al. (2009) confirmed a second gene *Foc0*_2_/*foc0*_2_ (flanked by TS47 and TA59 markers) on LG2 following analysis of two mapping populations CA 2156 × JG 62 and CA 2139 × JG 62. Earlier, this gene was discovered by Rubio et al. (2003). Likewise, Jendoubi et al. (2016) fine mapped the *Foc01/foc01* gene within an interval of 2 cM on LG5 using nearly isogenic lines (NILs). Of the 27 annotated genes, two candidate genes *LOC101514038* and *LOC101499491* are involved in disease resistance (Jendoubi et al., 2016). An SSR-based QTL analysis of F_2__:__3_ (C 214 × WR 315) elucidated two QTLs *FW-Q-APR-6-1* and *FW-Q-APR-6-2* on LG6 for *Foc1* resistance (Sabbavarapu et al., 2013; see Table 3). The SSR marker TA103 was used for introgression of *Foc1* from WR 315 to C 214 (Varshney et al., 2014). Previously, Gowda et al. (2009) located *Foc1* flanked with SSRs TA110 and H3A12 on LG2. The authors also mapped the *Foc2* (TA96-H3A12) and *Foc3* (TA194- H1B06y) on LG2. However, Jingade and Ravikumar (2015) reported one major QTL *GSSR 18-TC14801* on LG 1 for *Foc*1 resistance, which explained up to 71% phenotypic variation (PV). Subsequently, a major QTL *FW-Q-APR-2-1* on CaLG02 and two other minor QTLs *FW-Q-APR-4-1* and *FW-Q-APR-6-1* on CaLG4 and CaLG6, respectively were identified for resistance against *Foc1* and *Foc3* (Garg et al., 2018). Considering *Foc5*, monogenic/oligogenic nature has been established for resistance loci on LG 2 (Tekeoglu et al., 2000; Winter et al., 2000; Castro et al., 2010). Recently, by using SNP in combination with SSR markers the candidate genomic region on LG2 was narrowed down to 820 kb (Caballo et al., 2019b). The authors also suggested involvement of a putative candidate gene *LOC101511605* encoding CBL-interacting serine/threonine-protein kinase 8 in FW response.

**TABLE 3:** List of QTLs contributing to FW resistance in various grain legumes.

Crop	Mapping population size and type	QTL/gene	Type of marker and name	LG group	PV%	QTL method analysis	References
Chickpea	–	*H1* locus of *Foc 1*	CS27A(RAPD)	*–*	*–*	*–*	Mayer et al., 1997
Chickpea	C-104 × WR-315 (100 F_5_)	Single recessive gene (race 1 and race4)	CS-27_700_, UBC-170_550_ (RAPD)	*–*	*–*	*–*	Tullu et al., 1998
Chickpea	C. *arietinum* × C. r*eticulatum* (130, RIL)	races 4 and 5	STMS and a SCAR	*–*	*–*	*–*	Winter et al., 2000
Chickpea	131 (F_6_ RIL)	*foc-0*, *foc-4*, and *foc-5*	CS-27 (STS)	*–*	*–*	*–*	Tekeoglu et al., 2000
Chickpea	CA2156 × JG62 (RIL)	*Foc 01/foc 01*	OPJ20(600) (RAPD)	*–*	*–*	Maximum Likelihood method	Rubio et al., 2003
	CA2139 × JG62 (RIL)	*Foc 01/foc 01* and *Foc 02/foc 02*		*–*	*–*		
Chickpea	CA2139 × JG62 (RIL)	One gene resistance for Fusarium wilt race 0 (*Foc0*)	OPJ20(600) (RAPD) TR59(STMS)	LG3	*–*	*–*	Cobos et al., 2005
Chickpea	WR315 × C104	Three loci (race 2)	–			*–*	Kumar, 2006
	WR315 × K850						
	K850 × GW5/7						
Chickpea	WR-315 × C-104	*foc-3* gene	TA96 and TA27, TA196 (STMS)	*–*		MAPMAKER program	Sharma et al., 2004;
	100 F_7_	*foc-1* [syn. h(1)] and *foc-4*	CS27A (STS)				Winter et al., 2000;
			TA194 (STMS)				Gowda et al., 2009
Chickpea	CA2156 × JG62	Single gene (race 0)	OPJ20600 (RAPD)			*–*	Rubio et al., 2003
	CA2139 × JG62						
Chickpea	–	*Foc5*	TA59 and TA96 (SSR)	*–*	*–*	*–*	Cobos et al., 2009
Chickpea	F_9_	*Foc1*	H3A12 and TA110 (STMS)			*–*	Gowda et al., 2009
Chickpea		*Foc2*	TA96 and H3A12 (STMS)				
Chickpea		*Foc3*	H1B06y and TA194				
Chickpea	CA2139 × JG62 (RIL)	*Foc0*_2__/_*foc0*_2_	TA59 (STMS)	LG2	22*–*26	Interval mapping, multiple-QTL models (MQM)	Halila et al., 2009
	CA2156 × JG62 (RIL)						
Chickpea	–	*foc-5*	TA59 (STMS)	LG2			Castro et al., 2010
Chickpea	C214 × WR315	*FW-Q-APR-6-1 (Foc1)* and *FW-Q-APR-6-2 (Foc1)*	CaM1402 and CaM1101 (STMS)	LG6	10.4–18.8		Sabbavarapu et al., 2013
						QTLNetwork 2.0	
						Composite interval mapping	
Chickpea	JG62 × WR315, (94 RIL)	*Wilt 1* (race 1), *Wilt 2* (race 1)	TA27-TA59 (STMS)	LG2	16*–*36		Patil et al., 2014
			TA27-TA110 (STMS)				
Chickpea	C 214 × WR 315	Genomic region resistance for *foc1* and *foc3*	TR19, TA194, TAA60, GA16, TA110, and TS82	LG2	*–*	*–*	Varshney et al., 2014
Chickpea	K850 × WR315 (RIL, 140)	5 QTLs	GSSR 11-EST SSR 3	LG1	56–70		Jingade and Ravikumar, 2015
			TR 24-EST SSR 21				
			EST SSR 21-EST SSR 65				
			GSSR 18-TC14801				
Chickpea	CA2156 × JG62 (RIL, 80)	*Foc01/foc01*	H2I20 and TS43 (STMS)	LG5	*–*	Interval mapping	Jendoubi et al., 2016
	ILC3279 × JG62 (RIL, 115)	*LOC101514038* and *LOC101499491*	CaGM20820, CaGM20889				
	JG62 × ILC72 (RIL, 102)	Candidate genes					
Chickpea	Pusa 256 × Vijay	*Foc* 2	TA 37 and TA110	*–*		*–*	Pratap et al., 2017
Chickpea	JG62 × ICCV 05530	3 QTL (race 1), *FW-Q-APR-2-1*	TR19 and H2B061	CaLG02	6.6*–*31.5	QTL-IciMapping	Garg et al., 2018
		*FW-Q-APR-4-1, FW-Q-APR-6-1*	TA132 and TA46	CaLG04 and CaLG06			
		2 QTLs (race 3)	TA80 and CaM0594	CaLG02 and CaLG04			
		*FW-Q-APR-2-1* and *FW-Q-APR-4-1*	CKAM1256 and TS72				
Chickpea	WR315 × ILC3279 (RIL, 103)	*LOC101511605* (*Foc5*)	TA59, CaGM07922, SNPs	LG2	*–*	*–*	Caballo et al., 2019b
	ICCL81001 × Cr5-9 (RIL 88)						
Chickpea	Annigeri 1 × WR 315 (BC)	Genomic region conferring resistance against	TA59, TA96, TR19, and TA27	LG2		*–*	Mannur et al., 2019
	JG 74 × WR315 (BC)	*foc4*	GA16 and TA96				
Common bean	F_2_ and F_3_	One dominant gene for *Fop* race 4	–	*–*	*–*	*–*	Cross et al., 2000
Common bean	Belneb RR-1 A55	One major QTL	RAPD, U20.750	LG10	63.5	*–*	Fall et al., 2001
	RILs (76)						
Cowpea	CB5 × CB3, CB5 × 7964, F_1_, F_2_, and BC	One dominant gene (race 2), one dominant gene (race 3)	–	*–*	*–*	*–*	Rigert and Foster, 1987
Cowpea	California Blackeye 27 × 24-125B-1	*Fot3-1*	SNP 1_0860 and 1_1107 1_1484 and 1_0911	LG6	27.8	Kruskal*–*Wallis and interval mapping analysis	Pottorff et al., 2012
Cowpea	IT93K-503-1 × CB46	*Fot4*-*1* and *Fot4*-*2*. *Fot4*-*1*	-	LG3	*–*	Interval mapping	Pottorff et al., 2014
	CB27 × 24-125B-1						
	CB27 × IT82E-18/Big Buff						
Cowpea	A panel of 96 genotypes	17 significant MTAs for Fusarium wilt resistance	SNP 1_0075, 1_1111,1_1147, 1_0251, 1_0895, 1_0691, 1_0897, 1_0298, 1_0410, 1_0857, 1_0981, 1_1369, 1_0691, 1_0330, 1_1062, 1_0629, 1_0318, and 1_1504	LG1, LG3, LG4, LG5, LG6, LG8,LG9, LG10, and LG11	2–4	–*–*	Wu et al., 2015
Cowpea	F_1_, F_2_, and BC	–	SSR, C13−16			–*–*	Omoigui et al., 2018
Pea	Green arrow × PI179449	*Fw*	ACG:CAT_222(AFLP)		–*–*	–*–*	McClendon et al., 2002
			ACC:CTG(AFLP), Y15_1050(RAPD)	–*–*	–*–*		
Pea	–	*Fw*	–	LG3	*–*		Grajal-Martin and Muehlbauer, 2002
Pea	Shawnee × Bohatyr	*Fnw*		LG4	*–*		McPhee et al., 2012
	RILs (187)						
Pea	Lifter × Radley (393 RIL) and Shawnee × Bohatyr (187 RIL)	*Fw*	THO(CAPS marker)	LG3	70–*–*92	Single*–*factor ANOVA	Jain et al., 2015
			PRX1TRAP13(TRAP marker)				
			AnMtL6, Mt5_56				
Lentil	ILL 5588 × L 692-16-1(s), (RIL 86)	*Fw gene*	SSR59-2B, p17m30710	LG6	*–*	*–*	Hamwieh et al., 2005
Lentil	ILL5588 × L692–16−l(s)	*Fw gene*	OP−BH800 and OP−DI5500	*–*	*–*	*–*	Eujayl et al., 2006
	F_2:4_, RIL						
Pigeonpea	GSlxICPL87119,(254 F_2_)	One gene	RAPD (OPM03704 and OPAC11500)	*–*	*–*	*–*	Kotresh et al., 2006
	GS1 × ICP8863						
Pigeonpea	ICPL 20096 × ICPL 332	*C.cajan_03203*	SNP	LG2, LG11	*–*	Seq*–*BSA approach	Singh V. K. et al., 2016
	(RIL F_7_)	*C.cajan_07078*					
		*C.cajan_07124*					
		*C.cajan_02962*					
Pigeonpea	ICPB 2049 × ICPL 99050 (RIL)	*qFW1*.*1,qFW2.1*	SNP, S1_2827280–S1_4263752	LG1, LG2, LG3, LG4, and LG6	6.5*–*14	Composite interval mapping	Saxena et al., 2017
	ICPL 20096 × ICPL (332 RIL)	*qFW3*.*1,qFW4.1*	S2_16115010–S2_15580586				
	ICPL 85063 × ICPL 87119 (F_2_)	*qFW6*.*1,qFW11.1*	S3_18695411–S3_17153283				
		*qFW11*.*2,qFW11.3*	S4_597553–S4_1108184				
			S4_597553–S4_1108184				
			S6_22726005–S6_23553522				
			S11_37262913–S11_37133265				
			S11_43777543–S11_37133265				
			S11_20607023–S11_16809228				
			S11_4243778–S11_22408748				

In pea, genetic linkages of AFLP (McClendon et al., 2002), SSR (Loridon et al., 2005), SCAR (Okubara et al., 2005), and TRAP (Kwon et al., 2013) with FW was reported. Jain et al. (2015) identified a CAPS marker at 0.9 cM from the *Fw* locus on LG3, which could be effectively used for screening FW resistance in pea (see Table 3). Earlier, McPhee et al. (2012) reported two minor QTLs on LG 3 controlling resistance against *Fop* race 2.

In cowpea, SNP analysis of the population developed from CB27 × 24-125B-1 allowed identification of a 3.56 cM genomic region on LG1 for resistance to *Fot3-1* (Pottorff et al., 2012). These marker-trait associations (MTAs) explained up to 27.8% PV for the resistance. Furthermore, a comparative analysis between cowpea and soybean genomes suggested four candidate genes from the *Fot3-1* genomic region, which were related to leucine-rich repeat serine/threonine protein kinases (Pottorff et al., 2012). Likewise, two QTLs *Fot4-1* and *Fot4-2* imparting *Fot4* resistance were identified on LG 5 and LG 3, respectively (Pottorff et al., 2014). Synteny analysis between soybean and cowpea suggested a role for candidate genes underlying the *Fot4-1* and *Fot4-2* QTLs that code for TIR–NBS–LRR proteins and leucine-rich repeat serine/threonine protein kinases (Pottorff et al., 2014; see Table 3).

In lentil, Eujayl et al. (1998) tagged *Fw* locus controlling resistance to *Fol* at 10.8 cM from RAPD marker (OPK−15_900_). Subsequently, Hamwieh et al. (2005) reported *Fw* locus on LG 6 flanked by SSR59-2B and AFLP marker p17m30710.

In pigeonpea, different research groups have found significant MTAs and candidate genes for FW by using SSR (Patil et al., 2017a) and SNP markers (Singh V. K. et al., 2016; Saxena et al., 2017).

Whole genome sequence information in combination with high-throughput DNA marker technologies has divulged massive amounts of genome-wide markers to analyze MTAs in large germplasm sets for traits including FW resistance.

In common bean, GWAS on a diverse collection of 162 Portuguese accessions showed nine significant SNPson chromosomes Pv04, Pv05, Pv07, and Pv08 for *F. oxysporum* f. sp. *phaseoli* strain FOP-SP1 race 6. Authors also reported that the resulting candidate genes are engaged in phytoalexin biosynthesis, hypersensitive response, and plant primary metabolism (Leitão et al., 2020). Similarly, GWAS of 96 genotypes in cowpea revealed 11 significant MTAs (on LG 1, 3, 4, 6, 8, 9, 10, and 11) explaining 4% PV related to leaf damage traits and seven significant MTAs (LG 3, 6, 10, 11) explaining 9.7% PV related to vascular discoloration (Wu et al., 2015). Among the significant MTAs, two SNPs 1_0691 and 1_1369 showed close proximity to the QTL *Fot3-1* and *Fot4-2*, previously identified by Pottorff et al. (2012, 2014). A recent SSR-based association study of 89 pigeonpea lines phenotyped for 3 years in a wilt-sick field provided a set of six SSR markers, which were cross-validated in a biparental population segregating for FW (Patil et al., 2017a, b).

## Sequencing Based Approaches for Understanding the Plant-Wilt Interactions in Grain Legumes

### Whole Genome Sequencing of Host and FW Causing Pathogen: New Insights Into the Plant Defense System Against FW

Availability of whole genome sequence information in chickpea (Varshney et al., 2013), common bean (Schmutz et al., 2014), cowpea (Lonardi et al., 2019), pea (Kreplak et al., 2019), and pigeonpea (Varshney et al., 2012) could allow identification of the candidate gene(s)/the genomic regions controlling disease resistance. Concurrently, draft genome assemblies of *Fusarium udum* F02845 (Srivastava et al., 2018) and *Foc* (*Foc-38-1*) and *Fop* (*Fop-37622*) (Williams et al., 2016) have shed new light onto virulence-related genes that enhance our current understanding of pathogenicity of FW and evolution of the host-pathogen interaction in the legume species. Further, comparative genomics of *Fusarium* species could also elucidate the host specific gene(s), effector gene(s) and the sequence conservation across legume-infecting isolates and other *Fusarium* spp. (Williams et al., 2016). Additionally, construction of fungal pangenome could offer deeper insights into novel pathogen effectors and how they defeat host plant resistance rapidly (Badet and Croll, 2020). Thus, growing refinements in deep sequencing chemistry have paved the way for whole genome re-sequencing (WGRS) of global legume germplasms for capturing the large structural variations (SVs) including the copy number variations and presence-absence variations controlling various traits of economic importance including disease resistance (Varshney et al., 2013; Thudi et al., 2016). In pigeonpea, a comparison of whole genome sequence information of FW-resistant genotypes (ICPL 87119, ICPL 20097, ICP 8863, and ICPL 99050) and FW-susceptible genotype (ICPB 2049) in combination with Seq-BSA of the resistant and susceptible bulks (ICPL 20096 × ICPL 332) revealed four candidate genes including *C.cajan_03203* (Singh V. K. et al., 2016). These identified markers/candidate genes could be deployed for breeding FW resistance in pigeonpea. Genome-wide analysis using sequencing data of wilt responsive genotypes may help in pinpointing haplotypes responsible for resistance against multiple FW races, thus providing scope for gene pyramiding.

## Functional “Omics” Studies to Delineate Host Genes Imparting FW Resistance

Prior to the discovery of digital transcriptome profiling, expressed sequenced tags (ESTs), cDNA-AFLP, and cDNA-RAPD were largely employed to find the gene(s) participating in the plant defense mechanisms and plant-pathogen interactions (Wise et al., 2007; Ashraf et al., 2009; Gupta et al., 2009; Gurjar et al., 2012; Xue et al., 2015).

In common bean, cDNA-AFLP analysis of FW-resistant and susceptible genotypes revealed differential expression of 423 transcript derived fragments (TDFs), of which 98 TDFs had annotated functions in signal transduction, protein synthesis and processing, RNA and energy metabolism, defense and stress responses (Xue et al., 2015). Furthermore, q-RT-PCR analysis confirmed FW-responsive expression of 19 candidate genes in CAAS 260205 (resistant) and BR 130 (susceptible) genotypes. Some important candidate genes viz., *CBFi28*, *CBFi43* (ubiquitin protein), *CBFi45* (poly-ubiquitin protein), *CBFi76* (peroxidase), *CBFi54*, *CBFi58* (calcium dependent protein kinase), *CBFi83*, and *CBFi171* (NBS-LRR) had abundant expression in the resistant genotype (Xue et al., 2015).

In recent years, RNA-sequencing has enabled genome-wide surveys of transcriptomes to identify FW responsive candidate genes and their biological roles with greater precision and higher resolution (Li C. Y. et al., 2012; Kohli et al., 2014). Transcriptome analysis of four chickpea cultivars JG 62, ICCV 2, K 850, and WR 315 allowed several important “large effect” SNPs and Indels in the genomic regions controlling FW resistance (Jain et al., 2015). The underlying genomic region containing these SNPs and indels was predicted to be associated with defense related activity. Caballo et al. (2019a) functionally validated the genomic region controlling *Foc* (race 5) resistance in chickpea from resistant and sensitive NILs developed from the cross ILC 3279 × WR 315. Differential gene expression analysis at 24 h post inoculation (hpi) suggested two known candidate genes *LOC101499873* (encoding chaperonin) and *LOC101490851* and three novel candidate genes (*LOC101509359*, *LOC101495941*, and *LOC101510206* encoding MADS-box transcription factor, MATE family protein and serine hydroxymethyl-transferase, respectively) to be related to defense activity against FW. Likewise, nine genes viz., *LOC101503802*, *LOC101505941*, *LOC101506693* had significantly higher expression in FW sensitive NIL at 48 hpi than the FW resistant NIL (Caballo et al., 2019a). Transcriptome analysis of JG 62 and WR 315 in response to FW (race 1) infection uncovered abundance of differentially expressed transcripts related to various TFs, cellular transporters, sugar metabolism contributing to activate defense signaling against FW in chickpea (Gupta et al., 2013a; see Table 4). Further, network analysis also provided greater insights into the role of genes associated with defense components (MAP kinase, serine threonine kinase, etc.), reactive oxygen (superoxide dismutases, glutathione reductase, thioredoxin reductase, etc.), ATPase (myo-inositol phosphate, carboxylate synthase, etc.), significantly participating in the defense signaling against FW in chickpea (Gupta et al., 2013a). Similarly, differential expression was obtained by transcriptome profiling of chickpea genotypes JG 62 (*Foc* susceptible) and Digvijay (*Foc* resistant) for genes that are involved in lignification, hormonal homeostasis, plant defense signaling, reactive oxygen species (ROS) homeostasis, R-gene mediated defense in response to host-pathogen interaction (Upasani et al., 2017). An integrated analysis of transcriptome and metabolome data from the root samples of control and *FOP* infected seedlings in common bean demonstrated that pathogen establishment occurs after 24 h of infection, which is accompanied by timely induction of the defense mechanism. The study reinforced the proposition that the *FOP* defense system in common bean requires contributions from defense-related proteins such as glycosylphosphatidylinositol-anchored proteins (GPI-APs), signaling pathways mediated by hormones like salicylic acid, jasmonate and ethylene, and flavonoid biosynthesis pathway.

**TABLE 4:** Exhaustive list of various DEG and candidate genes contributing to FW resistance in grain legumes.

Crop	Genotype name	Differentially expressed genes/candidate gene	Function	Platform used	References
Chickpea	JG62 and WR315	6272 ESTs related to cell signaling and transcription and RNA processing and modification, cellular transport and homeostasis, hormone responses, cellular redox and energy metabolism, defense, and stress responsive genes	Defense mechanism, cellular metabolism and hormone signaling	RNA blot analysis	Ashraf et al., 2009
Chickpea	JG62 and WR315	Redox signaling genes such as redox regulatory respiratory burst oxidase homolog F (RBOHF), thioredoxin 3 (TRX3), cationic peroxidase 3 (OCP3), flavodoxin-like quinone reductase 1 (FQR1), iron superoxide dismutase 1, NADH cytochrome b5 reductase (CBR), Fe (II) oxidoreductase 7 (FRO7), Genes related to intracellular transportation *ABC transporter* like gene, polyol transporter gene, translocase, heavy metal transporter (detoxifying protein) (FRS6), bZIP, homeodomain leucine zipper, MYB, helix loop helix, zinc finger (CCHC type), and heat shock family protein, sucrose synthase (SUS4), β-amylase (BAM1), serine threonine kinase (CDKB1.1), and vacuolar ATPase (TUF)	Defense activity, ROS activity, and sugar transportation, sugar metabolism	qPCR	Gupta et al., 2013a
Chickpea	ICC4958	car-miRNA008 targets the chalcone synthase miR2118 and car-miR5213 targeting defense gene encoding Toll/Interleukin-1 receptor-nucleotide binding site-leucine-rich repeats miR2111 targets a Kelch repeat-containing F-box protein	Flavonoid biosynthesis involved in defense mechanism	Illumina (NGS) qPCR	Kohli et al., 2014
Chickpea	Digvijay and JG62	Glutamate dehydrogenase-GDH, glutamate synthase Glutamine synthase and asparagine synthase Methionine synthase and AdoMet synthetase CCoAMT, CHS, CHI, iso-flavone 40-O-methyltransferase, IFS, and IFR	Nitrogen mobilization and methionine metabolism, lignin and phytoalexin biosynthetic pathways	qPCR	Kumar et al., 2016
Chickpea	Digvijay and JG62	Chitin synthase VII (*Chs7*) G protein β subunit gene Mitochondrial carrier protein (*Fow1*) Xylanases gene, pectate lyases gene, polygalacturonases gene	Involved in establishment of pathogen in the host plant	qPCR	Upasani et al., 2016
Chickpea	Digvijay and JG62	3816 DEGs	Lignification, hormonal homeostasis, plant defense signaling, ROS homeostasis, R-gene mediated defense	LongSAGE	Upasani et al., 2017
Chickpea	NILs—RIP8-94-5 resistant R/RIP8-94-11 susceptible (S)	Upregulation of LOC101509359, LOC101495941, LOC101510206 genes in resistant NILs and upregulation of LOC101503802, LOC101505941, LOC101506693, LOC101507659, LOC101509037, LOC101510206, LOC101510544, LOC101501552, and LOC101502928 candidate genes in susceptible NILs	Encodes MADS-box transcription factor, encodes protein related to multidrug and toxic compound extrusion (MATE) family, encodes for a serine hydroxymethyl- transferase	qPCR	Caballo et al., 2019a
Chickpea	JG62 and WR315	PR10, pectinesterase, Aquaporin, ATP synthase, mtr3, exosome, immunity associated differentially expressed genes, cystatin and DnaJ, pectinesterase and xyloglucosyl transferase, actin and profilin-like genes, cytochrome P450, cytochrome P450 monooxygenase, and peroxidase	Immunity, ATP, ROS activity, cell wall remodelers, cytoskeleton related function	qPCR, Microarray analysis	Ashraf et al., 2018
Common bean	BRB130 and CAAS260205	122 transcript-derived fragments (redox-related genes, hormone response genes, transport-related genes, defense and stress response-related, signal transduction genes development and cytoskeletal organization). Upregulation of candidate genes viz., CBFi28,CBFi43,CBFi45,CBFi76,CBFi56, CBFi63, and CBFi122 CBFi54, CBFi58, CBFi83 and CBFi171 in resistant genotype	Signal transduction, hormonal response, defense and cellular metabolism, etc.	qPCR, cDNA-AFLP analysis	Xue et al., 2015
Common bean	Liyun No. 2	8269 downregulated genes, 13,771 upregulated genes Upregulated candidate genes *PHAVU_007G070400g*, *PHAVU_004G134300g, PHAVU_011G042100g*, *PHAVU_008G232600g, PHAVU_007G185300g.* Downregulated candidate genes *PHAVU_003G141800g*, *PHAVU_007G0495001g*, and *PHAVU_007G236300g* down regulation of UDP-glucuronic acid decarboxylase and cellulose synthases high accumulation of pectate lyases, pectin methylesterase inhibitors (PMEI), pectin methylesterases (PME), and Polygalacturonases genes, genes responsive to hormone signaling	Structural defense Defense related proteins Hormones signaling pathways Energy metabolism and nitrogen mobilization Flavonoid biosynthesis pathway	Illumina (NGS)	Chen et al., 2009

Further, to explore the role of micro RNAs (miRNAs) contributing to FW resistance, RNA-seq analysis of ICC 4958 uncovered known as well as novel miRNAs (car-miRNA008 targeting the chalcone synthase gene) involved in FW resistance in chickpea (Kohli et al., 2014). Among the identified miRNAs, miR530 showed 17-fold high expression, whereas miR156_1 and miR156_10 had slightly higher expression in response to FW infection (see Table 4).

### Proteomics and Metabolomics to Elucidate Plant Defense Mechanism Against FW in Grain Legumes

A proteomics approach allows for the unraveling of the various proteins engaged in host-pathogen interaction and their role in defending the host plant against pathogen attacks (Castillejo et al., 2015; Kumar et al., 2016). Significant roles for myriads of proteins in host-pathogen interactions have been suggested either during the establishment of the pathogen in the susceptible host plant or protecting the host plant from pathogen invasion (Rep et al., 2002; Berrocal-Lobo and Molina, 2008; Mehta et al., 2008; Castillejo et al., 2010; Palomares-Rius et al., 2011; Gunnaiah et al., 2012). These proteins included chitinases, xylem proteinases, β-1,3-glucanases, proteinase inhibitors, pathogenesis-related (PR) proteins, leucine rich-repeat proteins, proline-rice glycoproteins, cellulose synthases, ankyrin repeat containing protein, thaumatin-like protein PR-5b, syntaxins to subtilin-like proteases in various plant species in response to FW infection (Yang et al., 1997; De Ascensao and Dubery, 2000; Li T. et al., 2012; Castillejo et al., 2015; Kumar et al., 2016; Silvia Sebastiani et al., 2017). In the host plant several enzymes viz., glutathione S-transferases, peroxidases, peroxiredoxin, uinone oxidoreductase, copper amine oxidase, caffeic acid O-methyltransferase, chalcone synthase, chalcone isomerase, isoflavone reductase, phenylalanine ammonia lyase, etc., show change in response to FW invasion (Klessig et al., 1998; Garcia-Limones et al., 2002; Gupta et al., 2013a; Kumar et al., 2016). However, limited information is available in grain legumes on the participation of anti-fungal proteins especially for FW (Palomares-Rius et al., 2011; Scarafoni et al., 2013; Kumar et al., 2016). To establish roles of proteins in disease development or prohibiting pathogen attack for disease progression in pea, Castillejo et al. (2015) performed proteomic analysis using two-dimensional electrophoresis (2-DE) and mass spectrometry (MALDI-TOF/TOF), and the study found 53 proteins engaged in the plant’s response to *Fop* race 2 infection. These proteins were found to affect carbohydrate and energy metabolism (viz., fructokinase-like protein, beta-amylase, phosphoglucomutase, cytoplasmic), nucleotide and amino acid metabolism (viz., apyrase S-type, adenosine kinase/copper ion binding), signal transduction and cellular process (viz., chalcone O-methyltransferase, 4-3-3-like protein), redox and homeostasis (viz., short-chain alcohol dehydrogenase SAD-C, short-chain alcohol dehydrogenase A), defense (endochitinase A2, beta-1,3-glucanase), and biosynthetic process (viz., NADPH: isoflavone oxidoreductase, glutamate decarboxylase) (Castillejo et al., 2015). In chickpea, the role of various defense-related proteins was observed in restricting FW infection in the genotypes Digvijay (FW resistant) and JG 62 (FW susceptible) (Kumar et al., 2016). Several ROS activating enzymes viz., glutathione peroxidase, glutaredoxin, glutathione S-transferase, ascorbate peroxidase, peroxiredoxin were abundant in Digvijay as compared to JG 62. Likewise, the genotype Digvijay was able to restrict FW pathogen attack than FW susceptible cultivar JG 62 due to the abundance of PR proteins (Kumar et al., 2016). Thus, proteomics could further illuminate our understanding of the unknown proteins involved in various signal transduction pathways for inducing host innate immunity against FW attack in grain legumes. Concurrently, this approach could also enable us to discover the novel pathogen effectors that drive the arms race between host plants and pathogens.

Like proteomics, a metabolomics approach has greatly advanced our understanding about various metabolites, hormonal crosstalks, and signaling molecules that participate in plant defense mechanisms against FW pathogenesis in crop plants including grain legumes (Gupta et al., 2010; Kumar et al., 2016). The various metabolites produced in response to FW include sugars like hexokinase, glucose-6-phosphate, sucrose synthase, trehalose, invertase, β-amylase, etc. (Morkunas and Ratajczak, 2014). These sugars play a key role in plant resistance against pathogen attacks by serving as substrate for supplying energy, causing oxidative burst and ROS generation, enhancing lignification of cell wall, and acting as signaling molecule in concert with various phytohormones to induce plant innate immunity (Morkunas et al., 2005, 2007; Nikraftar et al., 2013; Morkunas and Ratajczak, 2014).

Besides sugars, the other important metabolites that are implicated in FW pathogen attack and entry into host plant include various amino acids, organic acids (pyruvate, lactate, acetate, etc.), nucleotides and their derivatives, antioxidants, phytoalexins (lignans, pisatins), polyphenols, phenolic acids (monomers of lignin), calmodulins, flavonoids, lipids, and phenylpropanoids (Klessig et al., 1998; Rolland et al., 2002; Garcia-Limones et al., 2009; Kumar et al., 2015, 2016; Bani et al., 2018a, b). Several plant hormones that serve as the essential signaling molecules in regulation of host defense response against FW infection are ethylene, salicylic acid, and jasmonic acid (Kunkel and Brooks, 2002). The plant immunity or plant defense response mediated by these phytohormone is defeated or suppressed by the attacking pathogen through production of toxins or effectors (Ma and Ma, 2016).

Metabolite profiling of common bean in response to *FOP* demonstrated participation of UDP-glucuronic acid decarboxylase, cellulose synthases, and pectate lyases, amino acids, glycosyl phosphatidyl inositol-anchored proteins, and various phytohormones (salicylic acid, jasmonate, and ethylene), polyphenols, anthocyanins, flavanones, and flavones during host plant, and *FOP* interaction. Similarly, an abundance of various proteins contributing in glycolysis and TCA processes, defense related metabolites (endo beta-1,3-glucanase, chitinases, caffeic acid O-methyltransferase, and caffeoyl-CoA O-methyltransferase), phytoalexins (genistein, luteolin, quinone), flavonoids, isoflavonoids, and phenolic compounds was observed in Digvijay than in JG 62 (Kumar et al., 2016). However, significant decrease in various amino acids and sugars viz., sucrose and fructose in susceptible cultivar allows FW pathogens to invade and promote disease development (Kumar et al., 2016). Therefore, further advancements in metabolomics could enable elucidation of intricate network of various signaling molecules and hormonal crosstalk contributing to FW resistance in grain legumes.

A comparison of the different studies that analyzed changes in plant transcriptomes, proteomes and metabolomes in response to FW infection reinforces the role of chitinases, PR proteins, ROS activating enzymes, phenolic compounds, flavonoids, phytoalexins in imparting wilt resistance (Castillejo et al., 2015; Kumar et al., 2016; Upasani et al., 2017; Bani et al., 2018a, b). These studies also highlight the significance of molecules that participate in cellular metabolism including carbohydrate, protein, nucleotides (Castillejo et al., 2015; Xue et al., 2015; Kumar et al., 2016) and signaling pathways involving MAP kinase, serine threonine kinase and various phytohormones (Gupta et al., 2013a, b; Xue et al., 2015).

## Future Prospect for Breeding for FW Resistance in Grain Legumes

### Focus of Phenomics Capturing Host Pathogen Interaction

The declining cost of genotyping and accumulation of huge genotyping data have allowed pinpointing the targeted genomics regions and the underlying causative gene(s)/genomic regions for a variety of important traits including FW resistance. However, linking of this genomic information with the phenotype still remains a daunting task due to complexity of G × E interactions (Araus and Cairns, 2014). Current state-of-the-art high throughput phenotyping (HTP) approach has the potential to bridge the genotype-phenotype gap for various complex traits (Fiorani and Schurr, 2013; Araus and Cairns, 2014). Recent advances in high-resolution imaging platforms and sensor technologies have revolutionized our capacity to investigate plant disease interaction, screening of disease resistant lines and identifying plant disease at large scale and large field (Lowe et al., 2017; Shakoor et al., 2017; Singh et al., 2018). Several HTP approaches including field-based remote sensing, 3D scanning, unmanned aerial vehicles system in association with multispectral and thermal cameras, RGB based imaging, fluorescence imaging to hyperspectral image sensing are worth mentioning, and are routinely employed for precise understanding of plant-pathogen interaction, detecting early stage disease symptoms and assessing disease severity (Mahlein et al., 2012; Römer et al., 2012; Mahlein, 2016; Lowe et al., 2017; Zhang et al., 2019). Given the cumbersome techniques of identifying disease infected plants and monitoring of plant disease symptoms both manually and visually that may delay preventing disease progression, early detection of disease symptoms through various sophisticated imaging techniques could assist in taking early preventive measures for restricting disease progression and crop yield loss (Mahlein, 2016; Lowe et al., 2017; Ghosal et al., 2018). Among the various fluorescence-based imaging analysis techniques used, the chlorophyll fluorescence imaging technique estimating F_v_/F_m_ remains a reliable phenotyping technique for monitoring plant-pathogen interaction and disease severity with greater precision (Berger et al., 2007; Bauriegel et al., 2011; Rousseau et al., 2013). Monitoring changes in leaf surface temperature of FW responsive genotypes through an infra-red system allowed identification of FW resistant and susceptible genotypes in pea (Rispail and Rubiales, 2015) and in *Medicago truncatula* (Rispail et al., 2015). Likewise, multi and hyperspectral imaging was used for early prediction of disease onset, differentiating healthy and diseased plants, quantifying disease infection and assessing disease severity in various plant species (for details see Lowe et al., 2017; Shakoor et al., 2017). Similarly, high resolution thermal and hyperspectral imaging approaches were used to detect early wilt of olive caused by *Verticillium dahliae* in a large acreage (Calderón et al., 2015). However, disease prediction accuracy completely depends on the curation and interpretation of acquired hyperspectral imaging data (Singh et al., 2018). Therefore, to improve prediction accuracy of various plant diseases, currently machine learning and deep learning (convolutional neural network and artificial neural networks) approaches have been combined with the hyperspectral imaging data (Mohanty et al., 2016; Sladojevic et al., 2016; Ghosal et al., 2018; Singh et al., 2018). Further interpretation of these images could allow us to identify disease with higher precision and accuracy and also assist in proper assessment of disease severity. Thus, in the era of “Crop breeding 4.0 driven by the big data,” HTP will be an integral component of genotype, phenotype, and environment-based decision-making models (Wallace et al., 2018; Jiang et al., 2019).

### Novel Breeding Techniques for Designing FW Resistant Grain Legumes

Grain legume cultivars with an enhanced level of FW resistance have been bred using conventional breeding approaches for many decades. However, this approach is time consuming, and demands (i) sufficient genetic variation in the breeding material and (ii) greater manpower for hybridization and handling of segregating population (Yin and Qiu, 2019). A continuous supply of FW-resistant varieties in response to evolving FW pathogens under changing climate demands adoption of efficient breeding technologies (Meuwissen et al., 2001; Gao, 2018; Ghosh et al., 2018).

Explosion of SNP data thanks to the high throughput genotyping platform has offered a great opportunity to adopt genomic selection (GS) in crop plants. GS is used to predict the genomic estimated breeding value of untested individuals using genome-wide marker data. The genotypic and phenotypic information of “training population” is used to train prediction models (Meuwissen et al., 2001; Jannink et al., 2010; Desta and Ortiz, 2014). This approach has been employed for developing disease resistant genotypes in wheat and its scope for breeding disease resistant genotypes has been discussed elsewhere (Poland and Rutkoski, 2016). Genome-wide predictions are yet to be employed for disease resistance in grain legumes.

Speed breeding (SB) or rapid generation advancement presents another promising means to reduce the crop generation time and accelerate the breeding program (Ghosh et al., 2018). This technology has allowed for the recovery of six generations per year in various crops including wheat, barley, chickpea, pea (Ghosh et al., 2018; Hickey et al., 2019). When combined with MAS, SB may dramatically accelerate the screening of the breeding populations against disease or targeted introgression of loci controlling resistance to susceptible genotypes.

**FIGURE 2:**
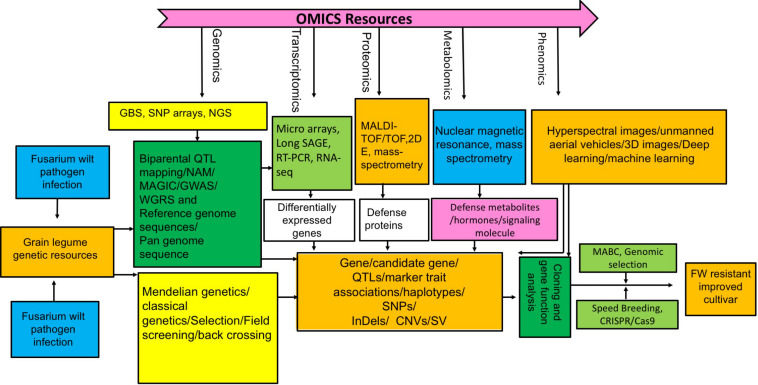
Integrated breeding, genetics, and “omics” scheme illustrating how to combat FW resistance in grain legume.

To circumvent the cumbersome process of trait manipulation in plants, CRISPR/Cas9 based genome editing technology is revolutionizing plant biology and breeding by precise modification of target gene sequence using customized nucleases (Voytas, 2013; Sander and Joung, 2014; Wang et al., 2014; Langner et al., 2018). This technique has been successfully employed to improve plant resistance against various bacterial and fungal diseases (Li T. et al., 2012; Wang et al., 2014, 2016; Chandrasekaran et al., 2016; Nekrasov et al., 2017; Peng et al., 2017; Zhang et al., 2017; Langner et al., 2018). In future this technique could be harnessed for improving FW resistance in grain legumes. An integrated approach involving various omics technologies and novel breeding schemes for future designing of FW resistant grain legumes has been depicted in Figure 2.

## Conclusion and Prospects

Severity and frequency of disease occurrence has seen a considerable rise in the wake of changing global climate, thus jeopardizing grain legume production worldwide. Breeding for FW resistance is a key breeding objective of crop improvement programs in grain legumes. Sourcing novel variations of FW resistance from unexploited CWRs and landraces needs greater attention to strengthen the genetic base. In parallel, pyramiding of different resistant gene(s) by adopting both standard backcrossing and DNA marker-aided approaches could expedite breeding of resistant cultivars. Advances in genomic technologies along with increasing genome sequence information could deepen knowledge about the resistant candidate genes/haplotypes to better breed FW-resistant grain legumes. Likewise, functional genomics could allow discovery of candidate loci, their biological functions and the molecular mechanisms underlying host-pathogen interactions. Importantly, emerging HTP phenotyping could illuminate the spatio-temporal aspects of host-pathogen interaction. Targeted and rapid manipulation of genomic loci responsible for FW resistance in grain legumes could be achieved with adoption of newer techniques like GS, SB, CRISPR/Cas9. An efficient combination of these new approaches paves the way for a steady stream of resistant legume cultivars that yield higher in increasing disease scenarios.

## Author Contributions

UCJ conceived the idea and wrote the manuscript with AB. UCJ developed the draft of the manuscript. AB and SKP contributed sections on omics technologies. SP contributed section on the FW infection and their effects on major grain legumes. AB edited the manuscript. All authors have read and approved the final manuscript.

## Conflict of Interest

The authors declare that the research was conducted in the absence of any commercial or financial relationships that could be construed as a potential conflict of interest.

## Footnotes

1 http://www.indexfungorum.org/

2 http://www.mycobank.org/
